# Engaging students and faculty: implications of self-determination theory for teachers and leaders in academic medicine

**DOI:** 10.1186/1472-6920-13-151

**Published:** 2013-11-11

**Authors:** Jeffrey M Lyness, Stephen J Lurie, Denham S Ward, Christopher J Mooney, David R Lambert

**Affiliations:** 1 Office of Academic Affairs, University of Rochester Medical Center, 601 Elmwood Avenue, Box 706, Rochester, NY 14642, USA; 2 Offices for Medical Education, University of Rochester Medical Center, 601 Elmwood Avenue, Box 601, Rochester, NY 14642, USA; 3 Foundation for Anesthesiology Education and Research, and Department of Anesthesiology, University of Rochester Medical Center, 601 Elmwood Avenue, Box 604, Rochester, NY 14642, USA

**Keywords:** Motivation, Medical education, Academic leadership

## Abstract

**Background:**

Much of the work of teachers and leaders at academic health centers involves engaging learners and faculty members in shared goals. Strategies to do so, however, are seldom informed by empirically-supported theories of human motivation.

**Discussion:**

This article summarizes a substantial body of motivational research that yields insights and approaches of importance to academic faculty leaders. After identification of key limitations of traditional rewards-based (i.e., incentives, or 'carrots and sticks’) approaches, key findings are summarized from the science of self-determination theory. These findings demonstrate the importance of fostering autonomous motivation by supporting the fundamental human needs for autonomy, competence, and relatedness. In turn, these considerations lead to specific recommendations about approaches to engaging autonomous motivation, using examples in academic health centers.

**Summary:**

Since supporting autonomous motivation maximizes both functioning and well-being (i.e., people are both happier and more productive), the approaches recommended will help academic health centers recruit, retain, and foster the success of learners and faculty members. Such goals are particularly important to address the multiple challenges confronting these institutions.

## Background

A course director, creating a new medical student course covering a large amount of required material, is warned by colleagues about the students’ poor attendance at lectures and seeming interest in learning only what will be on the examination.

The Dean’s Office receives complaints from faculty members that the medical school’s promotions criteria seem to be unclear and inequitably applied, leading them to feel powerless and disengaged from the process.

Medical educators frequently face difficulties motivating learners to achieve educational goals. Whether the specific concern is attendance, participation, or burnout, the question is raised: how do we motivate our students or residents not only to meet requirements, but to become fully engaged with the excitement of learning? Similarly, other academic health system leaders often encounter resistance from faculty asked to meet performance targets, comply with regulatory standards, or otherwise engage successfully with organizational missions. In this article, we summarize a substantial body of motivational research that yields insights and approaches of use to educators and academic faculty leaders. We begin by considering the limitations of traditional incentives. We then summarize key findings from self-determination theory, a comprehensive theory of motivation demonstrating the importance of fostering intrinsic, or autonomous, motivation. This theory leads to specific recommendations about approaches to engaging autonomous motivation. We conclude with examples that apply these recommendations in academic health centers.

Self-determination theory is of course only one of many scientific approaches to human motivation, many of which may be relevant to educators and other academic leaders [1]. We have focused on self-determination theory because it has been supported and extended by more than a thousand experimental and observational studies from many research groups [2-4]; moreover, everyday application of the theory is aided by its strong face validity — its concepts lend themselves to recommendations that make intuitive sense. While this research has been summarized elsewhere [2], and prior publications have considered its applicability to teaching and curricular design [1,5-9], here we will emphasize the systemic and administrative application of self-determination theory by leaders in academic medicine attempting to engage students or faculty members.

## Discussion

### Why not just reward the behaviors we want?

Perhaps the most common approach to influencing behavior is the use of direct tangible incentives, e.g., externally imposed rewards or punishments ('carrots and sticks’). Indeed, research on operant conditioning [10-12] has shown that incentives can effectively shape and modify behaviors. Most faculty and students have considerable experience within systems of positive and negative reinforcers. However, such tangible reward systems have at least three important limitations:

**Unintended consequences.** In their efforts to earn incentives, people often avoid non-incentivized behaviors (including other tasks that also may be important). Or they may achieve incentivized goals by taking shortcuts including, for some, unethical methods. In other words, incentives may produce the desired outcomes at the cost of a range of other undesirable outcomes [13]. For example, an attempt to improve student attendance at lectures by offering a grade boost if at least a certain number of students attend may result in the attendees rarely exceeding the minimum number specified. Similarly, attempts to improve clinical care by pay for performance can lead to a range of undesired consequences including physician resentment [14], poorer care in areas not directly incentivized [15], and possibly decreased value placed on other activities such as teaching and scholarship [16].

**No pay, no play.** Once a behavior has been incentivized by a direct tangible reward, people are actually *less* likely to perform that behavior after the incentive is removed. This is so even for activities initially perceived as fun or pleasurable [13,17,18]. This phenomenon is exemplified by individuals turning a longstanding hobby into a paid vocation; what formerly was fun now feels like 'work,’ unlikely to be pursued spontaneously unless it generates income. Furthermore, income above a basic threshold does not lead to increased happiness, a finding also true of monetary gifts or accumulation of purchases [19,20].

**Inhibition of creative problem solving.** While incentives do increase desired behaviors if the tasks are straightforward, incentives actually produce *poorer* performance on tasks that require flexible, creative problem-solving or other high-level cognitive processes. This finding has been replicated under many experimental conditions [3,13,21-23]. For example, groups asked to solve the 'candle problem,’ a task requiring novel, 'out of the box’ thinking, take longer to solve the problem if they are promised a financial reward [24].

Why do incentives have such important limitations and liabilities? From the perspective of self-determination theory, the problems with incentives are understood as the consequences of *extrinsic motivation*. In other words, incentives make the motivation feel imposed by others, as opposed to feeling driven from within (*autonomous motivation)*. We now turn, then, to consider self-determination theory and its implications for academic leaders.

### Overview of self-determination theory

While excellent reviews of self-determination theory have appeared for social scientists [25,26] and lay audiences [27,28], here we will briefly summarize key concepts in contexts more directly relevant to medical educators and faculty leaders.

Self-determination theory is a comprehensive theory of human behavior supporting “our natural or intrinsic tendencies to behave in effective and healthy ways” [4]. A key, empirically validated cornerstone of the theory is that supporting three basic psychological needs [3,26,29] engages one’s motivation from within, producing desirable benefits for learning, behavior, and well-being. These needs are as follows:

**Autonomy.** People want to have a sense of choice, to believe that they are exercising free will. It is important to recognize that, in this context, autonomy does not necessarily mean doing things alone. People often choose to do things in concert with others; it is the sense of choice, of feeling volitional, that is paramount.

**Competence (mastery).** Put simply, people like to feel that they are good at what they do. Some have referred to this concept as 'mastery’ [30-32], a reasonable term as long as it does not imply the necessity of reaching the highest possible level of proficiency. It is the growing sense of mastery at a task that engages motivation from within, driving the arduous reflective practice required to develop expertise [25,33].

**Relatedness (purpose).** People need to feel connected to other people, as noted for millennia by humanists as well as scientists [34-36]. This sense of connectedness may be felt on a direct interpersonal level. It also may be fostered by relating to a group, or to ideals or goals held by a group (including a society or culture); thus, relatedness also has been described as 'sense of purpose’ [28].

Fulfilling these basic human needs fosters what is known as autonomous motivation. (To be clear, this is not an all-or-none phenomenon; rather, there are degrees of relative autonomy, as extrinsic motivation may become internalized to a greater or lesser extent [3]). Supporting autonomous motivation maximizes functioning and well-being [22,37,38]; this has been particularly well-demonstrated among a range of students, for whom autonomy-supportive teachers lead to better learning and achievement, greater flexibility and creativity, more positive emotionality, and higher rates of retention [39,40]. Put more colloquially, meeting these three basic needs leads to people being both happier and more productive, goals that are obviously desirable among trainees and faculty alike. Fostering autonomous motivation among clinicians also may increase their support of patients’ autonomous motivation in clinical practice [5,41], with potential benefits in modifying patient health behaviors [42-45].

### How to support autonomous motivation

Given the importance of engaging autonomous motivation, the following describes specific approaches to fostering the three fundamental needs, summarized in Table 1. These approaches are highly complementary, as satisfying one need often supports the others [25]. The goal is to enhance autonomous motivation, leading to increased learner and faculty engagement; thus, using the framework of Kusurkar et al. [46], motivation is by turn both a dependent variable, i.e., a desired outcome of our recommendations, and an independent variable, i.e., producing the ultimate desired outcomes of engagement, morale, and productivity [47].

**Table 1:** Approaches to foster the three basic psychological needs as articulated by self-determination theory

**Basic psychological needs**		
**Autonomy**	**Competence**	**Relatedness**
Take others’ perspectives	Set an optimal level of challenge	Acknowledge feelings and convey empathy
Provide choices	Support the skills development necessary to meet the posed challenge	Create structures to foster individual connections
Provide a meaningful rationale when choices cannot be offered	Give meaningful feedback framed positively toward the achievement of competence	Create structures to foster group and community connections
Minimize controlling words		

### Supporting autonomy

#### ***Take their perspective***

Asking people for their perspective supports their sense of autonomy [48]. Seeking such input also may usefully convey empathy and foster their sense of relatedness to the 'authorities’. Examples of taking perspective are the inclusion of students in curricular evaluation processes, and using faculty surveys (such as the Association of American Medical Colleges’ Faculty Forward project [49]) to shape faculty development initiatives.

#### ***Give choices***

Having more than one option to choose from naturally fosters a sense of choice. Providing choices may involve creating additional options. For example, an educational program may offer 'selectives’ in place of single required courses, or faculty may have the choice of promotion along any of several career paths ('tracks’). The educational modality of problem-based learning (PBL) emphasizes self-directed learning [50] and gives students choices [51] in both group discussion topics and individually-researched topics to present to the group; while we are unaware of empirical research directly assessing the effects of PBL on perceived autonomy, PBL does increase student interest and enjoyment [52,53]. Institutional leaders also may support autonomy by focusing on the choice aspects of seemingly nonnegotiable demands. For example, an advisor can help a mentee reframe a required activity as an aspect of the mentee’s chosen career path, and thus as part of the journey to which they have committed.

#### ***When alternative choices are not possible, provide a meaningful rationale***

By understanding the reasons for a lack of alternatives, people may adopt the sole 'option’ as their own choice, i.e., from the perspective of self-determination theory, they may internalize what might otherwise be taken as an extrinsic motivation [54] (“given the circumstances, it makes sense so I choose to go along with it”). Providing rationale also fosters a sense of relatedness with the leaders, recognizing that the leaders’ intentions align with one’s own. For example, due to changed Public Health Service (PHS) regulations, our medical school recently amended its policy to require more frequent faculty reports of a broader range of outside remunerations. While faculty had no choice in the matter, School leadership communicated detailed explanation about its rationale, including the process that led to the new PHS regulations and the implications of non-compliance (e.g., loss of PHS grant funding).

#### ***Minimize controlling words***

Not surprisingly, words and intentions such as “ought”, “must”, and “should” decrease a sense of autonomy [39]. While their use in some contexts is unavoidable, it is important to use them sparingly and only when necessary, to minimize undermining perceived autonomy.

### Supporting competence

#### ***Set an optimal level of challenge***

While unrealistically high expectations undermine a sense of competence, expectations that are too low fail to produce the satisfaction that occurs with developing a sense of mastery. (The concept of a 'just-right’ degree of difficulty also is consistent with other theories of motivation, including the seminal work of Atkinson [55] later incorporated into expectancy-value theory). Educators setting an optimal level of challenge must have a deep understanding of the learners’ skill set while determining the educational goals ('know your audience’). Similarly, school leaders function most effectively by knowing well the capabilities of their faculty members, setting the 'productivity bar’ appropriately. Of course, individual variability often makes it difficult to create an optimal level of challenge for all.

#### ***Support the skills development necessary to meet the posed challenge***

Armed with an understanding of learner or faculty abilities, leaders can increase motivation by providing the right combination of experiences, conditions, and tools to enable the development of the skills required to master the task at hand. In the above example of faculty members’ reporting outside remuneration, our school leaders created online survey software to both prompt and facilitate collection and reporting of changes in external income and of travel within 30 days, as required by the new PHS regulations. Communications to faculty included information about the new system, thereby giving faculty members the ability to accomplish a task that at first may have triggered feelings of incompetence (“how am I supposed to remember and keep track of all that?”). The success of this approach is evident not only in faculty compliance, but by the relatively muted objections to what otherwise might have been perceived as an onerous burden.

#### ***Give meaningful feedback framed positively toward the achievement of competence***

It is widely understood that negative feedback undermines a sense of competence and thus may be experienced as demotivating [13,56]. However, positive feedback that is merely praise also can reduce autonomous motivation [57], a seemingly paradoxical finding that can be understood as the consequences of the feedback in effect becoming an extrinsic (i.e., controlling) motivator. Positive feedback can foster autonomous motivation if it is not administered in a controlling fashion, and if it is meaningful, i.e., if it contains substance helping the individual genuinely recognize her/his competency gains and identify the next steps to further mastery [13,48,58,59]. Faculty leaders may provide frameworks for giving such feedback to those involved in teaching, e.g., structured guides for student feedback both 'mid-way’ and at the end of a course, or templates for conducting departmental annual reviews of each faculty member, both further supported by faculty development seminars on giving feedback and using the guides or templates.

### Supporting relatedness

#### ***Acknowledge feelings***

As long recognized by communication skills training in medical education [60,61], observations about another’s emotional state convey empathy and build a sense of trust and reciprocity, e.g., “you look sad”, “it’s clear this is a frustrating situation”, or “you look like the 'light bulb’ really came on!” Therefore, acknowledging feelings and conveying empathy support autonomy [39] by addressing the need for relatedness.

#### ***Create structures to foster individual connections***

Academic leaders foster relatedness by facilitating the formation of interpersonal relationships with representatives of the institutional culture. For medical students, availability of faculty advisors, or frequent exposure to faculty members in small group settings such as PBL, foster more personalized connections than typically occur in whole class lecture settings. For faculty, formalized mentoring programs provide one structure within which lasting interpersonal relationships can form. As another faculty example, a welcome/orientation session for new faculty members may incorporate activities explicitly designed to initiate individual connections, such as a 'speed-meeting’ session in which participants are arbitrarily placed in a series of dyadic encounters, each telling the other something about both work and outside interests.

#### ***Create structures to foster group/community connections***

High-functioning organizational units typically feel like a cohesive whole to their members. Institutional leaders can help ensure that the culture of their groups function this way for existing members, while also welcoming new members and supporting their acculturation in becoming part of the group. For students this may occur both at the level of their entire class — using curricular and extracurricular activities to foster the students’ sense of themselves as a cohesive class — and in small groups in classroom, laboratory, or clinical settings. As an example, in one study students reported intra-group relationships as the biggest motivators in their PBL group work [62]. Similarly, for faculty the approaches may include large-group events, including purely professional activities (e.g., departmental faculty meetings, 'Town Halls’ with the Dean) and social events, together with activities in smaller collectives (e.g., laboratories, divisions) to create a sense of belonging to groups in which one knows all of the members.

### Examples

The following scenarios exemplify many of these principles, also illustrating how the three basic needs may be fulfilled in highly complementary fashion. They demonstrate how actions taken by course or administrative faculty leaders may increase autonomous motivation of students or faculty, leading to greater engagement of these constituents.

#### ***Example 1: medical students***

A 2nd-year medical student course enjoys strong student engagement. The large amount of required course material (neurosciences, pathology, pharmacology) does not allow students many choices of what to learn. However, student autonomy and competence are intended to be fostered by 'planned redundancy’ of course content across lectures, PBL small groups, and small group laboratories, allowing individuals to emphasize learning via whichever modality they wish. As well, the PBL method gives the students considerable choice regarding topics to cover in their discussions and individually researched mini-presentations to their group, and student perspectives are regularly elicited and acknowledged. Course teachers explicitly tell the students how the course content in each session relates to prior and upcoming material, and how it applies to clinical practice, thus providing a meaningful rationale for the topics covered. Relatedness among the students and with course faculty is built by small group interactions in PBL and labs. Lectures are intended in part to foster a sense of connection with the course leaders and among the class as a whole, using personal stories and humor to create a sense of a community of learners with a common mission. Thus the course appears to foster the students’ sense of autonomy, competence, and relatedness, resulting in them being more productive (learning the material, participating actively in class) and happier (class morale and student feedback about the course).

#### ***Example 2: faculty***

Responding to faculty concerns about School promotion processes seeming unclear and inequitably applied, leaders from the Dean’s Office extensively revise the promotions guidelines. Faculty sense of autonomy is supported by leaders noting that the revisions were undertaken in response to faculty concerns and feedback (i.e., taking their perspective), and by detailed explication of the goals of the revisions (i.e., providing rationale). Competence is supported by the greater specificity of the revised guidelines, providing faculty with better tools to help them achieve successful promotions outcomes. Relatedness is fostered by engaging with faculty about the revisions, collectively as a School community (e.g., using a web-based faculty newsletter), and on more personal bases by joining departmental faculty meetings and seeking individual dialogues as faculty members provide input and ask questions. While it is too early to know the longer-term impact of these activities, thus far faculty feedback about the process and the new guidelines has been very positive.

### Limitations

We have not offered a detailed critique of the science of self-determination theory, as more extensive reviews of the theory and alternative approaches to motivation may be found elsewhere [1-3,9,25,26,63]. What we have provided is explicit consideration of the theory’s systemic and administrative application to students and faculty by leaders in academic health centers. We also note that our recommendations, while fully consistent with self-determination theory and with the evidence base and prior recommendations for teaching students [5-7,39], are largely yet to be tested with medical faculty members. Also, to be clear, we do not believe that extrinsic incentives can be entirely abolished. Indeed, ensuring the attainment of standards for graduation, licensure, promotion, and other aspects of quality and achievement are essential to fulfilling our public obligations regarding medical and scientific practice. Moreover, an institution cannot move toward defined goals in a coordinated, efficient, and effective manner if the actions of its constituents are left entirely free to the fulfillment of individual wishes. Leaders must keep sight of non-negotiable goals, such as those related to regulatory mandates as well as larger institutional priorities, thus requiring a balance of supporting autonomous motivation with using incentives judiciously.

## Summary

Medical educators and faculty leaders can benefit by applying principles from self-determination theory to their teaching of students and their oversight of faculty spanning clinical, educational, research, and community missions. The theory also provides a helpful conceptual framework for understanding the results of satisfaction surveys [64]. The challenges confronting institutions, with rapidly changing expectations across all missions in the face of severe fiscal constraints, require attracting, retaining, and engaging talented faculty [65]. There are clear opportunities for empirical investigation to directly test the outcomes of specific approaches in increasing the perceived autonomy of faculty, as to date there has been little empirical research on the application of self-determination theory in academic health centers other than in education. But existing work strongly suggests that academic health center leaders engaged in strategic planning should design and frame their efforts so as to engage their constituents’ autonomous motivation as much as possible. Those that do so, those who are best able to foster a sense of autonomy, competence, and relatedness in learners and faculty, may be the most successful in harnessing their collective productivity to meet the challenges faced by our institutions.

## Competing interests

The authors declare that they have no competing interests.

## Authors’ contributions

JML and CJM reviewed the literature. JML wrote the initial draft of the manuscript. All authors participated in active discussions about the application of the research literature to specific examples from academic health centers. All authors read the manuscript, provided edits and critiques, and approved the final manuscript.

## Pre-publication history

The pre-publication history for this paper can be accessed here:

http://www.biomedcentral.com/1472-6920/13/151/prepub

## Previous presentations

Some of the ideas in this paper were presented at the June 2011 annual meeting of the Association of Directors of Medical Student Education in Psychiatry, Savannah, GA, and at seminars at the University of Rochester Medical Center.
